# P-2287. Are Solid Organ Transplant or Hematopoietic Stem Cell Transplant Recipients at Greater Risks of Unfavorable Outcomes of *Pneumocystis* Pneumonia?

**DOI:** 10.1093/ofid/ofae631.2440

**Published:** 2025-01-29

**Authors:** Poramed Winichakoon, Hanan Albasata, Susan M Poutanen, Seyed M Hosseini-Moghaddam

**Affiliations:** University Health Network, Chiang Mai, Thailand; Dubai Academic Health Corporation, Dubai, Dubai, United Arab Emirates; University Health Network and Sinai Health Network, Toronto, Ontario, Canada; University Health Network, Chiang Mai, Thailand

## Abstract

**Background:**

Solid organ transplant (SOT) and hematopoietic stem cell transplant (HSCT) recipients with *Pneumocystis* pneumonia (PCP) appear to be at risks of adverse outcomes and mortality with limited data.

**Methods:**

In this retrospective cohort, we compared the PCP manifestations, including clinical presentation, laboratory data, imaging studies, treatment and outcomes between SOT and HSCT recipients diagnosed with PCP at University Health Network, Toronto, Canada, from January 2011 to January 2022. We measured a composite outcome including 21-day ICU admission and/or 28-day all-cause mortality following PCP diagnosis. Using multivariable logistic regression analysis, we estimated adjusted odds ratio (aOR) to determine the concurrent effects of covariates.

**Results:**

During the accrual period, a total of 32 (55.2%) HSCT (20 allogeneic, 11 autologous, and one cord-blood transplant recipients) and 26 (44.2%) SOT (10 kidney, 8 liver, 5 lung, and 3 heart recipients) fulfilled the PCP diagnosis criteria. Notably, chronic steroid treatment was more common in SOT than in HSCT recipients [24 (92.3%) vs 13 (40.6%), p = 0.001]. The median time of post-transplant PCP diagnosis was 488 days with interquartile range (IQR) 90-481 in the HSCT group, and 1,453 (IQR 38-3,909) days in the SOT group. In comparison, dyspnea [25 (96.2%) vs 23 (71.9%), p = 0.015], desaturation [16 (72.7%) vs 12 (38.7%), p = 0.015], and pleural effusion [8 (30.8%) vs 2 (6.3%), p = 0.014] were more frequent in the SOT group. We observed a higher fungal load [11,450 (IQR 2,207-191,750) vs 10,295 (IQR 2,405-21,325) copies/mL, p = 0.043] and occurrence of the composite outcomes [13 (50%) vs 3 (9.4%), p = 0.001) in the SOT than the HSCT group. In the multivariable analysis, SOT recipients (aOR 7.1, IQR 1.3-37.4), patients received chronic steroid treatment (aOR 1.3, IQR 0.2-6.6), required supplemental O2 at the initial presentation (aOR 1.8, IQR 0.3-10.4), and had pleural effusion (OR 10.4, IQR 1.5-70.8) were significantly at the risk of composite outcome.

**Conclusion:**

SOT recipients appear to be at a greater risk of ICU admission and mortality than HSCT recipients. SOT or HSCT recipients with pleural effusion or requiring supplemental O2 on admission, particularly if receiving chronic steroid therapy, are particularly at risk of severe outcomes.

**Disclosures:**

All Authors: No reported disclosures

